# Impacts of the medical arms race on medical expenses: a public hospital-based study in Shenzhen, China, during 2009–2013

**DOI:** 10.1186/s12962-022-00407-7

**Published:** 2022-12-26

**Authors:** Paicheng Liu, Xue Gong, Qianhui Yao, Qiong Liu

**Affiliations:** 1 School of Public Administration, Southwestern University of Finance and Economics, Chengdu, China; 2 Department of East Asian Studies, University of Vienna, Vienna, Austria; 3 School of Politics and Public Administration, Guangxi Normal University, No.15, Yucai Road, Qixing District, Guilin, Guangxi People’s Republic of China

**Keywords:** Medical arms race, Medical expense, Public hospital, Medical service, China

## Abstract

**Background:**

Has the medical arms race (MAR) increased healthcare expenditures? Existing literature has yet to draw a consistent conclusion. Hence, this study aims to reexamine the relationship between the MAR and medical expenses by the data from public hospitals in Shenzhen, China, during the period of 2009 to 2013.

**Methods:**

This study’s data were collected through panel datasets spanning 2009 to 2013 from the Shenzhen Statistical Yearbook, Shenzhen Health Statistical Yearbook, and annual reports from the Shenzhen Municipal Health Commission. The Herfindahl–Hirschman index and hierarchical linear modeling were combined for empirical analysis.

**Results:**

The MAR’s impact on medical examination fees differed during the inpatient and outpatient stages. Further analysis verified that the MAR had the most significant impact on outpatient examination fees. Due to the characteristics of China’s medical system, government regulations in the healthcare market may consequently accelerate the MAR among public hospitals. Strict government regulations on the medical system have also promoted increased medical examination costs to some extent. Once medical service prices are under strict administrative control, only drug and medical examination fees are the primary forms of extra income for hospitals. After the proportion of drug fees is further regulated, medical examinations will then become another staple method to generate extra revenue. These have distorted Chinese public hospitals’ medical fees, which completely differ from those in other countries.

**Conclusion:**

The government should confirm that they have allocated sufficient financial investments for public hospitals; otherwise, the competition among hospitals will transfer the burden to patients, and especially to those who can afford to pay for care. A core task for public hospitals involves providing safer, less expensive, and more reliable medical services.

## Background

Has the medical arms race increased healthcare expenditures? Existing literature has yet to draw a consistent conclusion. Examinations of the rapid growth of China’s medical expenses have predominantly focused on external factors, but these are less common among hospitals. Essentially, China has rapidly implemented universal medical insurance and steadily improved the quality of its health services [1], but the accompanying increase in medical expenses has also caused anxiety. Regarding the factors affecting the growth of medical expenses, existing studies have covered aging, socioeconomic factors, economic growth, government health investments, and medical payment modes [2–6]. As such increased medical expenses occurred in hospitals, we consider it essential to analyze the growth mechanism of medical expenses from the perspective of the medical arms race.

Competition among hospitals has led to an expansion of medical equipment that has also been called the “medical arms race (MAR)” [7]. Hospitals preferred to purchase high-tech medical devices under this circumstance in order to flaunt their superior skills for handling sophisticated clinical examinations and treatments, which is known as MAR based on relevant literature as a competitive tactic for attracting patients [8–10]. Most previous studies have considered the MAR’s positive impacts, such as increasing hospitals’ quantities of both patients [11] and income [12]. However, the MAR has also had a negative impact, and the Chinese government’s involvement may be essential in promoting the healthcare system [13]. Public hospitals in China account for 89% of all beds and 92% of hospital admissions [14], providing more than 90% of the nation’s health services [15]. Nonetheless, the public hospitals in pilot schemes have not received sufficient funding from the government to distribute any profits to their staff [16]. Hospitals must gradually and increasingly compete to acquire more benefits.

Existing studies demonstrate a positive correlation between MAR and medical expenses [17, 18]. On the one hand, MAR is essentially a competition for quality health services [19]. Due to the information asymmetry between physicians and patients, it is difficult not only to measure medical quality, but also for patients to make rational decisions in clinical practice. High-tech medical equipment has become a signal that marks higher-quality hospital medical services [11]. Therefore, hospitals with high-tech equipment can attract more and better medical staff, and thus, appeal to more patients to choose and visit their facility [20, 21].

On the other hand, investments in high-tech equipment result in high costs. The payback burden of equipment may trigger supplier-induced demand [22] leading to increased medical expenses [23]. Additionally, medical operations will increase the labor cost of staff. Under the pressure of these two costs, hospitals tend to provide more expensive services to increase revenue [24]. In the highly competitive medical market, the sunk costs caused by MAR are also irreversible. Under these circumstances, hospitals will not adopt a strategy of lowering prices or reducing medical service supply, and therefore, MAR results in an overall increase in medical expenses [25].

Despite extensive evaluations conducted on the consequences generated by MAR, insufficient evidence was provided by empirical research in the field to clarify the factors of MAR which would further accelerate the increase of examination expenses and treatment expenses. For a long time, scholars have blamed external factors such as social and economic indicators for the growth of medical expenses in China overwhelmingly [2–6]. However, MAR provoked by the intense competition among hospitals seem to be ignored, which would generate a lack of analysis from the perspective of patients. In other words, when highlighting the adverse influences of MAR on the healthcare system, the profits of patients might be squeezed quietly as well.

Generally, it is still unclear as to which increases in medical expenses are caused by MAR. We address these issues by considering public hospitals in Shenzhen, China, as the research target. We then collected data on medical expenses spanning 2009 to 2013 and employed a panel regression model for our empirical analysis. This study’s implication is to not only effectively control the growth of medical expenses, but also improve the accessibility of medical services for patients, and especially those who cannot afford to pay for care. More importantly, this study will fill the research gap in the relationship between MAR and medical expenses.

## Materials and methods

### Data

This study’s data were collected through panel datasets spanning 2009 to 2013 from the Shenzhen Statistical Yearbook, Shenzhen Health Statistical Yearbook, and annual reports from the Shenzhen Municipal Health Commission. Data prior to 2009 were not included to omit macro-policies’ impacts on the research results. Specifically, data from this period were omitted because China’s New Medical Reform policy was launched in 2009. Data posterior to 2013 were not covered because the public data has been unavailable since then. But the sample capacity of the existing years is still enough to conduct the empirical analysis of this study. We employed public hospitals in Shenzhen as the research sample, with the hospital and year as the unit of analysis. This panel dataset includes 60 units and five time points, with 300 observations. Table 1 displays the variables’ measurements and data sources.

**Table 1:** Variable measurements and data sources

Variables	Measurements	Data sources
Medical expenses	Outpatient fees per visit	*Shenzhen Health Statistical Yearbook*
	Inpatient fee per capita	
Medical arms race	The total cost of medical equipment worth over 1 million yuan	*Annual reports from the Shenzhen Municipal Health Commission*
	The total amount of medical equipment worth over 1 million yuan	*Shenzhen Health Statistical Yearbook*
Medical resources	Number of physicians	*Shenzhen Health Statistical Yearbook*
	Number of nurses	
	Number of beds	
Government financial support	Government financial investments	*Shenzhen Health Statistical Yearbook*
	Government financial investments/total hospital revenue	*Shenzhen Health Statistical Yearbook*
Economic development of the district	GDP per capita in the district	*Shenzhen Statistical Yearbook*
hospital type	1 = General hospitals 0 = Specialized hospitals	*Shenzhen Health Statistical Yearbook*
Hospital grade	1 = Tertiary hospitals 0 = Secondary or primary hospitals	*Shenzhen Health Statistical Yearbook*
Healthcare market competition in the district	Herfindahl–Hirschman Index (HHI)	*Shenzhen Health Statistical Yearbook*
Aging rate in the district	Population over age 60 in the district/district population	*Shenzhen Statistical Yearbook*

We selected public hospitals in Shenzhen for two reasons. First, its thriving medical equipment industry has paved the way for MAR. With a robust healthcare system, the output value of Shenzhen’s medical equipment industry exceeded 40 billion yuan, accounting for as much as 8% of the medical equipment market worldwide [27, 28]. Second, as an economically flourishing city, Shenzhen is representative of China. With a permanent population of 13 million and 38,006 medical institutions, and 3.65 beds per 1000 people in 2018, Shenzhen has a developed medical system as well as the burden of substantial demand for medical services attributed to a large population, making it a typical city to be analyzed.

This study’s analysis only included public hospitals and excluded private hospitals because of the apparent disparities between these two kinds of hospitals in China. First, the broad expansion of private hospitals was driven by an unbiased market policy environment after 2001, but in China’s healthcare system, the majority of private hospitals are much smaller in scale than public hospitals [29], especially in terms of inpatient beds number [30]. In addition, public hospitals are far more competitive in terms of healthcare quality and health insurance reimbursements [31, 32]. Private hospitals have disadvantages for medical professionals [31]. More importantly, permanent urban residents valued private health care less [33], which is particularly obvious in metropolitan cities like Shenzhen.

Second, the types of private hospitals in Shenzhen are predominantly specialized medical institutions, akin to the current situation of the healthcare system nationwide [29]. However, private hospitals related to aesthetic medicine accounted for a large proportion in Shenzhen, which seems unsuitable to compare directly with patients who have rigid demand for medical services due to illness in public hospitals. Hence, the exclusion of private hospitals can reduce any analysis bias. Moreover, considering the collected panel dataset, we adopted hierarchical linear modeling (HLM) for empirical analysis. As both hospital- and district-level variables exist in this study, HLM can better analyze any variance in the hierarchical variables.

### Measures and model

#### Medical expenses and the medical arms race

Medical services consist of outpatient and inpatient care; therefore, we utilized the outpatient fees per visit and inpatient fees per capita to measure medical expenses. Subsequently, we observed that hospitals’ MAR participation primarily manifests in their investments in high-tech medical equipment [7, 34]. We referred to the calculation method in existing literature [12] to measure the MAR among hospitals by the amount and total cost of medical equipment worth over 1 million yuan.

#### Medical resources

Existing studies indicate that a positive correlation could exist between medical resources and medical expenses [35, 36]. The supplier-induced demand theory demonstrates that physicians with financial incentives may exploit asymmetric information to induce patient demand, thus leading to increased medical costs [37, 38]. Additionally, the target income theory further posits that increasing the supply of medical resources may create corresponding competition, leading to a decrease in the income among physicians. To achieve a target income, physicians may be inclined to provide excessive medical services to patients [39]. A previous empirical analysis also discovered that increasing the number of physicians creates over-diagnoses and treatments [40], thus remarkably increasing patients’ medical costs. Therefore, we included the supply of medical resources as a control variable in this study. Medical resources are measured by the number of physicians, nurses, and beds.

#### Healthcare market competition

Competition among hospitals may positively impact health spending growth [8]. We adopted the Herfindahl–Hirschman Index (HHI) to measure the degree of healthcare market competition, as this index is derived from economics measuring a region’s degree of industrial concentration. For this study, HHI means the degree of market competition among all hospitals within each district. Therefore, we calculated the HHI of medical services (outpatient visits) in every district in Shenzhen to measure the degree of healthcare market competition, with HHI values ranging from zero to one. A small HHI value indicates a high degree of healthcare market competition in the district, and vice versa. As Formula (1) indicates, X represents the total outpatient visits of all hospitals in a district, and Xi represents the outpatient visits of hospitalizations in that district:

1 $${\text{HHI}} = \mathop \sum \limits_{i = 1}^{N} \left( {X_{i} /X} \right)^{2}$$

#### Hospital type

Medical expenses may be higher in specialized hospitals than in general hospitals. As patients suffering from difficult, complex diseases tend to choose specialized hospitals than those with common diseases, intractable diseases are more complicated, generally leading to higher medical expenses [41]. Therefore, we included the hospital type as our control variable. Further, a value of one was assigned to represent general hospitals, and zero to represent specialized hospitals.

#### Other control variables

Other control variables included in the study are the government’s financial support for hospitals, the GDP per capita in the district, the proportion of the aging population in the district, and the hospital’s grade.

## Results

### Descriptive analysis

Table 2 presents the variables’ descriptive characteristics. In terms of medical expenses, the outpatient fees per visit were 145.61 yuan, with a maximum of 479 yuan, which was much lower than the average. The inpatient fee per capita was 5414.40 yuan, which may indicate that the inpatient fee was an important component of high medical expenses. The distribution of inpatient expenses among 60 public hospitals was evident, with a significant difference between the maximum and minimum. The minimum inpatient fee was zero, and this may relate to the fact that some secondary hospitals had no inpatient departments.

**Table 2:** Descriptive characteristics

Variables	Mean	S.D	Min	Max
Outpatient fee per visit (RMB)	145.61	75.188	42.266	479.514
Inpatient fee per capita (RMB)	5414.6	4680.702	0	32,652.564
Total amount of medical equipment worth over 1 million yuan	16.667	25.927	0	220
Total cost of medical equipment worth over 1 million (in ten thousand yuan)	42.993	6,216.026	0	414.28
Number of beds	322	343.836	0	2286
Government’s financial investment (in ten thousand yuan)	1764	5639.35	0	71,564
Hospital revenue (in ten thousand yuan)	2919.35	56.919	0	78,092.5
Government’s financial investment/hospital revenue	0.573	0.368	0	1.738
GDP per capita in the district (in ten thousand yuan)	13.776	9.572	2.538	75.562
Aging rate (over age 60) rate in the district	0.031	0.01	0.017	0.053
Herfindahl–Hirschman Index (HHI)	0.354	0.571	0.110	0.492

In terms of variables related to the MAR, on average 17 units of medical equipment were worth over 1 million yuan per hospital, with a maximum of 200 and minimum of 0. This reflects the substantial difference in the distribution of medical equipment worth over 1 million yuan among public hospitals. The total cost of medical equipment worth over 1 million yuan per hospital was 42.993 thousand yuan on average, and the standard deviation was also large, indicating that only a few public hospitals possess most of the high-priced medical equipment.

According to the correlation matrix in Table 3, a positive relationship could be observed between independent variables (the total cost of medical equipment worth over 1 million yuan and total amount of medical equipment worth over 1 million yuan) and dependent variables (outpatient fee per visit and inpatient fee per capita). Among independent variables, the total cost of medical equipment worth over 1 million yuan is significantly associated with the total amount of medical equipment worth over 1 million yuan. Thus, only one of them was included as the main independent variable and the other one was used for the robust test. Given the high standard deviation of the total cost of medical equipment worth over 1 million yuan, which might cause imprecise results, the total amount of medical equipment worth over 1 million yuan was chosen as the main independent variable for regression models.

**Table 3:** Correlation matrix

Variables	Outpatient fee per visit	Inpatient fee per capita	Total cost of medical equipment worth over 1 million	Total amount of medical equipment worth over 1 million yuan	Number of physicians	Number of nurses	Number of beds	Government’s financial investment	Hospital revenue	GDP per capita in the district	Aging rate in the district
Outpatient fee per visit	1										
Inpatient fee per capita	0.703*	1									
Total cost of medical equipment worth over 1 million	0.442*	0.461*	1								
Total amount of medical equipment worth over 1 million yuan	0.467*	0.397*	0.855*	1							
Number of physicians	0.213*	0.365*	0.841*	0.702*	1						
Number of nurses	0.258*	0.390*	0.859*	0.721*	0.982*	1					
Number of beds	0.349*	0.408*	0.860*	0.747*	0.933*	0.949*	1				
Government’s financial investment	0.227*	0.156*	0.351*	0.331*	0.250*	0.262*	0.378*	1			
Hospital revenue	0.254*	0.224*	0.531*	0.486*	0.450*	0.462*	0.567*	0.937*	1		
GDP per capita in the district	0.725*	0.521*	0.464*	0.458*	0.285*	0.339*	0.382*	0.223*	0.290*	1	
Aging rate (over age 60) rate in the district	− 0.108	− 0.075	− 0.027	− 0.033	− 0.078	− 0.120*	− 0.149*	0.026	− 0.018	− 0.135*	1

*p < 0.05

In addition, a high correlation was observed among the number of physicians, nurses, and beds, making it necessary to include only one of those to reduce bias caused by high correlation. In this research, medical resource, the control variable, was operationalized by the number of beds for the following reasons. First, due to strict regulations on the number of beds, the high number of beds normally indicates the high capacity of hospitals as well as the high number of nurses and physicians, but not vice versa [36, 37]. Hence, the number of beds is representative of operationalizing medical resources. Second, compared to the number of nurses and physicians, the correlation between the independent variable and the number of beds is smaller (less than 0.8). Even though the number of beds is still not lowly correlated to the independent variable, it is highly correlated to dependent variables and determined to be significantly associated with medical expenses according to regression models. Hence, the number of beds was still included as the control variable.

### Relationship between the medical arms race and medical expenses

According to Model 1 in Table 4, the total amount of medical equipment worth over 1 million yuan was positively associated with the outpatient fees per visit, indicating that investments in high-tech equipment may result in an evident increase in medical expenses. We also noticed that the total amount of medical equipment worth over 1 million yuan had a more significant impact on outpatient fees compared to inpatient fees. As high-tech medical services were mainly provided during inpatient care [20], the MAR’s different impacts on outpatient and inpatient fees seemed abnormal. To test the robustness of the MAR’s effect on medical expenses, we ran regression models with the total cost of medical equipment worth over 1 million yuan as the independent variable, the result of which turned out to be in line with the original models.

**Table 4:** Hierarchy of the linear regression for medical expenses

Variables	Model 1	Model 2	Model 3	Model 4
	Outpatient fee per visit	Inpatient Fee per Capita	Outpatient fee per visit	Inpatient fee per capita
Total amount of medical equipment worth over 1 million yuan	0.200** (0.001)	0.086* (0.048)		
Total cost of medical equipment worth over 1 million yuan			0.300*** (0.001)	0.095** (0.040)
Number of beds	0.000*** (0.000)	0.009 (0.007)	0.000 (0.000)	0.004 (0.008)
Government’s financial investment/total hospital revenue	− 0.012 (0.050)	2.279 (2.914)	− 0.010 (0.049)	2.187 (2.903)
GDP per capita in the district	0.000 (0.000)	0.000 (0.000)	0.000 (0.000)	0.000 (0.000)
Aging rate (over age 60) rate in the district	− 9.221 (9.785)	− 341.840 (574.678)	− 10.288 (9.339)	− 363.529 (561.070)
Healthcare market competition in the district	0.102*** (0.025)	1.307 (1.417)	0.111*** (0.024)	1.539 (1.421)
Hospital type	− 0.339*** (0.128)	9.350 (9.948)	− 0.372*** (0.129)	8.287 (9.858)
Hospital grade	0.260* (0.137)	23.552** (10.205)	0.239* (0.137)	22.689** (10.102)
Cons	1.448*** (0.348)	35.090* (20.894)	1.501*** (0.333)	36.434* (20.426)
Level 3—District				
Cons	− 1.337*** (0.309)	2.403*** (0.543)	− 1.422*** (0.329)	2.345*** (0.579)
Level 2—Hospital				
Cons	− 0.854*** (0.103)	3.548*** (0.099)	− 0.839*** (0.103)	3.540*** (0.099)
Level 1—Year				
Cons	− 1.624*** (0.046)	2.429*** (0.046)	− 1.655*** (0.046)	2.427*** (0.046)
N	300	300	300	300

***p < 0.001, **p < 0.01, *p < 0.05, ^+^*p* < 0.1

We further analyzed the abnormal result above by dividing both outpatient and inpatient fees into drug, examination, and treatment fees, then conducted a regression based on new dependent variables. As Table 5 indicates, the total amount of medical equipment worth over 1 million was only positively associated with outpatient examination fees. Therefore, the MAR had an evident influence on outpatient examination fees, but no significant impact on inpatient examination fees.

**Table 5:** Panel regression for medical expenses

Variables	Model 5	Model 6	Model 7	Model 8	Model 9
	Outpatient drug fee per visit	Outpatient examination fee per visit	Inpatient drug fee per capita	Inpatient treatment fee per capita	Inpatient examination fee per capita
Total amount of medical equipment worth over 1 million yuan	0.000 (0.001)	0.000** (0.000)	0.004 (0.017)	0.008 (0.019)	0.001 (0.001)
Number of beds	0.000 (0.000)	0.000 (0.000)	0.001 (0.002)	− 0.005* (0.002)	0.000* (0.000)
Government’s financial investment/total hospital revenue	− 0.001 (0.031)	− 0.026* (0.016)	1.519 (1.013)	− 0.350 (1.136)	0.003 (0.068)
GDP per capita in the district	0.000 (0.000)	0.000 (0.000)	− 0.000 (0.000)	0.000 (0.000)	0.000 (0.000)
Aging rate (over age 60) in the district	− 4.402 (6.963)	− 2.434 (1.578)	17.065 (158.525)	− 52.389 (151.848)	− 1.996 (6.302)
Healthcare market competition in the district	0.067*** (0.015)	0.009 (0.008)	0.492 (0.501)	0.415 (0.571)	− 0.015 (0.034)
Hospital type	− 0.185 (0.114)	− 0.044* (0.025)	9.194*** (2.360)	3.014 (2.186)	0.557*** (0.132)
Hospital grade	0.269** (0.118)	0.014 (0.026)	4.781*** (2.491)	5.159** (2.319)	0.428*** (0.133)
Cons	0.689*** (0.252)	0.285*** (0.057)	2.552 (5.682)	6.107 (5.423)	0.813*** (0.232)
Level 3—District					
Cons	− 1.905*** (0.457)	− 3.313*** (0.384)	1.348*** (0.364)	1.342*** (0.348)	− 1.536*** (0.276)
Level 2—Hospital					
Cons	− 0.927*** (0.099)	− 2.550*** (0.112)	2.061*** (0.107)	1.954*** (0.108)	− 0.784*** (0.100)
Level 1—Year					
Cons	− 2.118*** (0.046)	− 2.737*** (0.046)	1.393*** (0.046)	1.526*** (0.046)	− 1.294*** (0.046)
N	300	300	300	300	300

***p < 0.001, **p < 0.01, *p < 0.05, ^+^*p* < 0.1

The differential effects on outpatient and inpatient fees indicate that the MAR might induce over-examination. In China, payment for inpatient treatment is primarily based on diagnosis-related groups and the prospective payment system, which tightens the regulations involving the provision of high-tech medical services and medical costs’ growth. Unlike inpatient care, the reimbursement of outpatient visits is not restricted by these payment systems. Thus, over-examination is more likely to occur during outpatient visits than inpatient care.

## Discussion and conclusions

This study primarily revealed that the MAR was positively associated with medical bills; therefore, the MAR will result in higher medical costs. This view is consistent with previous studies’ conclusions [11, 12]. However, a further analysis demonstrated that the MAR’s effect on examination fees was more significant in the outpatient stage than in the inpatient stage. Previous scholars have observed that expanding hospitals’ use of more expensive medical equipment might lead to higher diagnostic examination expenditures [42]. Given this, we also considered that the MAR’s impact on examination fees differed during the inpatient and outpatient stages. As duplicated inpatient services may produce positive financial returns [20], total inpatient out-of-pocket expenses will increase substantially [43]. This conclusion broadens the interpretive scope of the MAR and medical expenses.

Higher medical costs may occur for the following reasons. First, the MAR can improve hospitals’ competitiveness. As medical services’ quality can be difficult to evaluate, the possession of high-tech equipment can be regarded as a signal of such quality [44]. Hospitals with high-value medical devices attract patients more easily and earn more medical income by providing advanced medical services [12]. Second, financial pressure caused by the investment in such equipment may force hospitals to provide excessive medical services to obtain extra income. Consequently, hospitals may tend to increase the demand for medical services but may also create increased waste from such resources due to the moral hazard [6].

Moreover, government regulations in the healthcare market may accelerate the MAR among public hospitals, or may inhibit competition among hospitals, which could further restrain the MAR. Competitive pricing is an effective strategy for increasing medical costs. From the mid-1980s to the mid-1990s, the MAR in the United States decelerated because hospitals’ competition became wasteful [45]. One possible reason was that expanding managed care encouraged hospitals to compete for selective contracting with insurance companies, which stimulated competitive pricing, and more importantly, encouraged hospitals to control costs and improve quality [46, 47]. Under these circumstances, the MAR required substantial investments and resulted in much higher medical costs; it was no longer a cost-effective strategy for hospitals to compete in the healthcare market [48]. The MAR was then suppressed and hospitals began competing by providing medical services of lower cost and higher quality.

However, China’s medical system differs, as its governmental involvement is essential in promoting healthcare systems [13], although Chinese government regulations on medical prices could also reduce competition among hospitals. As the prices of medical services are completely determined by the government [15], public hospitals cannot adopt countermeasures to control their prices to cope with their rivals. Moreover, the health insurance system covering all public hospitals could weaken the incentive to incur medical costs [26]. Unlike managed care and the MAR [48] in the United States, all public hospitals in China are covered by health insurance schemes, and thus, lack incentives to control medical costs [49]. As public hospitals were then unable and unwilling to price competitively, they joined the MAR, which allowed them to obtain income by attracting more patients and better compete in the healthcare market.

Second, strict government regulations on the medical system resulted in distorted medical fees [49] and promoted increased medical examination costs to some extent. While medical service prices are under administrative regulation, drug and medical examination fees are core sources of extra income for hospitals. After the proportion of drug fees is also regulated, medical examinations become a staple for hospitals to generate extra revenue, which transfers the burden to the patients who can afford to pay for care [26, 49]. A further effect is that this could destroy the medical system’s structure of expenditures [50]. Consequently, hospitals have a strong incentive to provide excessive medical examinations to gain extra income and lower the proportion of drug fees, leading to increased medical costs.

The Chinese government can control the growth of medical costs caused by the MAR by providing sufficient financial support for public hospitals; otherwise, it might be difficult for public hospitals to remain welfare-oriented. Nevertheless, the government has long been unable to ensure adequate financial investment in public hospitals [16]. Under financial pressure, public hospitals may be profit-motivated to employ several strategies to generate revenue, such as excessive examinations. Physicians’ control of medical services may then threaten hospitals’ financial health [41]. This may benefit physicians, but will increase the medical burden for patients, and especially for families with catastrophic medical expenses [4]. Therefore, the government should not only provide sufficient financial investments in public hospitals to reduce the incentives to earn medical income by overtreatment, but also encourage the provision of safer, less expensive, and more reliable medical services for all patients.

## Limitations

As this study was limited by the availability of data, it only focused on public hospitals in Shenzhen. However, different patterns of MAR and its relationship with medical costs may vary in other Chinese cities or other countries. Shenzhen’s experience may not necessarily be relevant in China’s less-developed provinces, especially those in the hinterland. Therefore, researchers should close this gap with samples from different cities or countries in the future. Besides, there are some other questions could not be answered by this research, such as the more specific causality between the MAR and medical expenses. Hence, qualitative study should be conducted to explore or examine the causality and mechanism.

## Data Availability

The datasets used during the current study are available from the corresponding author on reasonable request.

## Abbreviations

MAR: Medical arms race
HLM: Hierarchical linear modeling
HHI: Herfindahl–Hirschman Index
